# Activation of Stimulator of Interferon Genes (STING): Promising Strategy to Overcome Immune Resistance in Prostate Cancer

**DOI:** 10.2174/0109298673273303231208071403

**Published:** 2024-02-12

**Authors:** Mohammed Alnukhali, Omar Altabbakh, Ammad Ahmad Farooqi, Alan Pollack, Sylvia Daunert, Sapna Deo, Wensi Tao

**Affiliations:** 1 Department of Biochemistry and Molecular Biology, Miller School of Medicine, University of Miami, Miami, FL 33136, USA;; 2 Department of Radiation Oncology, Miller School of Medicine, University of Miami, Miami, FL 33136, USA;; 3 College of Medicine, Dr. Kiran C. Patel College of Osteopathic Medicine, Nova Southeastern University, Clearwater, FL 33759, USA;; 4 Institute of Biomedical and Genetic Engineering (IBGE), National Institute for Genomics and Advanced Biotechnology, Islamabad 44000, Pakistan;; 5 Leonard M. Miller School of Medicine, Clinical and Translational Science Institute, University of Miami, Miami, FL 33136, USA;; 6 The Dr. John T. McDonald Foundation Bionanotechnology Institute, University of Miami, Miami, FL 33136, USA

**Keywords:** STING pathway, radiotherapy, immune resistance, prostate cancer, cGAS-STING, autoimmune disease

## Abstract

Prostate cancer (PCa) is the most frequent and second-lethal cancer among men. Despite considerable efforts to explore treatments like autologous cellular immunotherapy and immune checkpoint inhibitors, their success remains limited. The intricate tumor microenvironment (TME) and its interaction with the immune system pose significant challenges in PCa treatment. Consequently, researchers have directed their focus on augmenting the immune system's anti-tumor response by targeting the STimulator of the Interferon Genes (STING) pathway. The STING pathway is activated when foreign DNA is detected in the cytoplasm of innate immune cells, resulting in the activation of endoplasmic reticulum (ER) STING. This, in turn, triggers an augmentation of signaling, leading to the production of type I interferon (IFN) and other pro-inflammatory cytokines. Numerous studies have demonstrated that activation of the STING pathway induces immune system rejection and targeted elimination of PCa cells. Researchers have been exploring various methods to activate the STING pathway, including the use of bacterial vectors to deliver STING agonists and the combination of radiation therapy with STING agonists. Achieving effective radiation therapy with minimal side effects and optimal anti-tumor immune responses necessitates precise adjustments to radiation dosing and fractionation schedules. This comprehensive review discusses promising findings from studies focusing on activating the STING pathway to combat PCa. The STING pathway exhibits the potential to serve as an effective treatment modality for PCa, offering new hope for improving the lives of those affected by this devastating disease.

## 1 INTRODUCTION

PCa continues to pose a significant public health concern, as it remains the second leading cause of cancer death in men. The estimated new number of PCa cases is about 288,300 in the United States alone [1]. It is challenging to combat the insidious PCa advance because of the TME [2, 3]. TME effectively disables the body’s defense mechanisms and provides fertile ground for cancer cells to thrive [4]. Even with the extensive expression of tumor-associated antigens, for example, PSMA and PSA, the activation of immune response is insufficient. Scientists have long been working on targeting malignancy with immunotherapy [5]. Autologous cellular immunotherapy has been attempted to be used to treat metastatic castration-resistant PCa (mCRPC) [6, 7]. Unfortunately, it has been met with limited response due to immune checkpoint inhibitors (ICIs) [8, 9]. This has prompted researchers to explore alternative immunotherapeutic options [10]. STING has emerged as a promising pathway in anticancer therapy [11, 12]. It stimulates the production of IFN, which activates cyclic Guanosine monophosphate - Adenosine monophosphate synthase (cGAS) promoting the anti-cancer immune response [13, 14].

Comprehending the mechanisms behind PCa immune resistance and the possibility of combining STING with other anti-PCa therapies is crucial to defeating PCa [15, 16]. This review offers a deeper insight into cancer immune resistance and highlights the potential of using the STING pathway to enhance cancer detection and elimination by the immune system. Innovative therapeutic approaches that harness the power of the immune system have the potential to revolutionize the field of cancer treatment.

## 2 IMMUNE RESISTANCE MECHANISMS IN PROSTATE CANCER

Researchers are constantly seeking innovative ways to characterize and combat cancer. One method is to classify tumors as “hot” or “cold” by considering tumor characteristics [17, 18]. “Hot” tumors typically elicit an immune response aimed at stemming their spread. In contrast, “cold” tumors are characterized by decreased tumor-infiltrating lymphocytes (TILs) associated with an increased immunosuppressive cell type [19, 20].

Unfortunately, PCa is categorized as a “cold” tumor due to the constellation of mechanisms that make up the immune repose (Table **1**) [21]. A possible explanation is that prostate-specific CD4 T cells do not successfully recognize the prostate gland [22]. One mechanism involves the suppression of anti-tumor and therapeutic responses due to the loss of IFN, which causes disease progression [23]. Furthermore, when T cells were collected from PCa tumor cells, they were found to be tolerant of the tumor antigens due to their inability to degranulate and produce IFN [24]. Basically, as compared to healthy prostate tissue, proinflammatory molecules, like IFN, that combat PCa are down-regulated [25]. The STING activation has been shown to be effective at enhancing the immune response to PCa. It triggers the production of IFN, expanding the T cell effect on the PCa TME [26].

### 2.1 Myeloid-Derived Suppressor Cells and Tumor Microenvironment in Prostate Cancer

The accumulation of an immunosuppressive population of Myeloid-derived suppressor cells (MDSC) within the tumor microenvironment (TME) has been shown to be associated with PCa tumor development and progression [27].

MDSCs are a subset of immature myeloid cells (IMCs) that exhibit potent immunosuppressive properties against T and natural killer (NK) cells. MDSCs differentiate into macrophages, granulocytes, or tissue-resident dendritic cells under normal conditions. These embryonic myeloid cells become activated to monocytes and neutrophils during an inflammatory response [28].

Over time, prostate tumor cells acquire genetic alterations that enable them to migrate from the primary tumor site to other anatomic sites and metastasize. In animal models, it was discovered that bone, a major metastatic site for advanced PCa in humans, contains substantial populations of MDSCs [29].

MDSCs were also found to be substantially elevated in the circulation of mCRPC patients, both in the untreated and docetaxel-treated groups compared to the control group, with CD4^+^ T-cell proliferation being significantly inhibited in both groups [30]. In addition, previous studies have demonstrated an upregulation in the production of chemokines that promote the recruitment of MDSCs to the PCa TME, while the expression of small molecules responsible for the recruitment of cytotoxic T lymphocytes (CTL) has decreased significantly [31]. This provides additional evidence that the PCa tumor microenvironment promotes the presence of these immune suppressor cells, resulting in a weakened antitumor immune response that could otherwise aid in disease progression control. STING activation in prostate cancer can stimulate the immune system, leading to the recruitment of immune cells such as T cells, natural killer cells, and dendritic cells into the tumor microenvironment. This influx of immune cells enhances the anti-tumor immune response, potentially leading to the recognition and destruction of cancer cells. However, prolonged STING activation can lead to the release of growth factors and other signaling molecules that promote angiogenesis and the formation of new blood vessels. This can provide the tumor with increased access to nutrients and oxygen, which can support tumor growth.

In summary, the activation of the STING pathway in prostate cancer can have both positive and negative effects on the tumor microenvironment. It can enhance the anti-tumor immune response, but prolonged or excessive activation may lead to chronic inflammation, angiogenesis, immunosuppression, and tissue remodeling, all of which can impact the course of the disease. The precise impact of STING activation on the tumor microenvironment may vary depending on the context and stage of the cancer.

## 3 STING PATHWAY IN CANCER

Cancer immunotherapy is hindered by insufficient IFN signaling and antigen presentation [37, 38]. Multiple studies have shown that the presence of IFN and activated CD8^+^ T cells in the TME correlated with positive outcomes on anti-tumor therapeutic modalities. IFN promotes the development and migration of antigen-presenting cells (APCs) [39]. IFN hinders cancer cell multiplication and enhances the expression of MHC-I, which is necessary for CD8^+^ T cell recognition [40, 41]. Knocking out IFNαR in CD8α+ dendritic cells (DCs) decreased antigen presentation and T cell priming. Mice with knocked-out IFN-α/β receptors did not have the capability to reject tumor cells [42]. STING pathway acts as a foreign DNA sensor that activates signaling to produce inflammatory cytokines, mainly IFN [43, 44]. STING has multiple effects on tumors, including modifying vasculature and augmenting adaptive immune response [45, 46]. In certain tumors, such as PCa, the expression of the STING pathway is inhibited [47]. Overturning the tumor inhibitory effect on the STING pathway has the potential to enhance the innate immune system's anti-tumor response.

STING pathway is an essential part of the immune response against malignant cells and infections [48]. The attachment of foreign DNA to the cGAS receptor activates the STING pathway producing cyclic GMP-AMP (cGAMP) [49]. The ER STING gets activated by the cGAMP [50]. ER STING turns on the Tank-binding kinase (TBK1), which phosphorylates interferon regulatory factor 3 (IRF3) and inhibitor of nuclear factor-κB (IκBα). IRF3 and IκBα lead to the phosphorylation of STING and IRF3 homodimers. These homodimers can penetrate the nucleus membrane to turn on the IFN transcription [51, 52]. One effect of the activation of STING and IFN production is the autophagy promotion. Autophagy works to eliminate foreign bodies by degrading cell membranes [53]. Furthermore, autophagy acts as a negative feedback regulator, ensuring the proper signaling and functioning of STING [54].

On the other hand, proinflammatory cytokines are induced by the phosphorylated IκBα. Proinflammatory cytokines include interleukin-6 (IL-6), tumor necrosis factor (TNF), and IFN. These pathways can be used to develop effective therapies that stimulate the immune system to fight cancer.

**Fig. (1):**
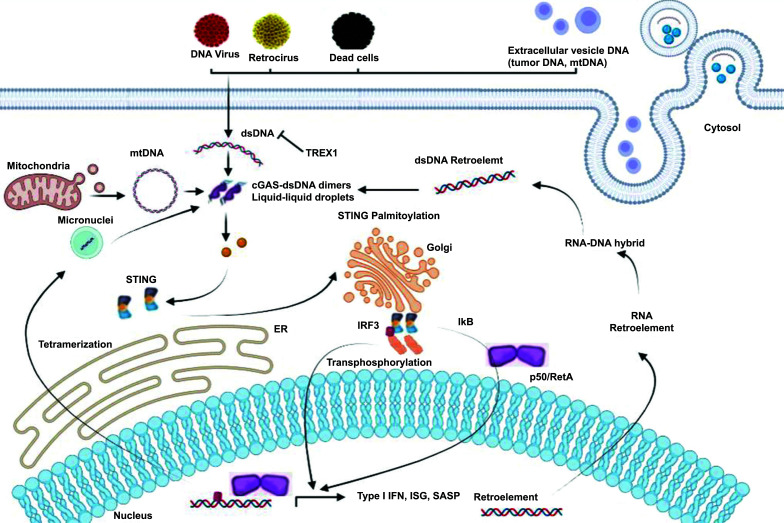
STING signaling pathway.

The intricate involvement of STING in the context of cancer is characterized by its multifaceted role. On one hand, short-term stimulation of STING emerges as a potent trigger for the immune system, fostering a remarkable efficacy in eliminating tumor cells through the immune antigen system. This transient activation, when finely tuned, harnesses the body's defense mechanisms to combat cancer. Conversely, when STING experiences excessive and sustained activation, a cascade of adverse effects is set in motion. This persistent stimulation leads to the generation of chronic inflammatory signals that can incite the release of various signaling molecules, such as cytokines, chemokines, and growth factors. These molecular signals, in turn, promote the formation of new blood vessels, a process known as angiogenesis. It is this sustained angiogenic drive that contributes to the progression and development of cancer, as the burgeoning network of blood vessels fuels the nourishment of malignant cells. Furthermore, extended activation of STING, beyond its initial beneficial phase, emerges as a double-edged sword. It can disrupt the host's internal balance, or homeostasis, and compromise the overall well-being of the individual. Notably, research has revealed that this prolonged activation of STING is implicated in the mediation of programmed cell death in B and T cells, ultimately leading to their demise. Paradoxically, this dual role of STING can also favor the proliferation of tumor cells, thereby fostering the progression of cancer. In this intricate interplay, the balance between short-term immune activation and the potentially detrimental consequences of chronic stimulation underscores the pivotal role of STING in the complex landscape of cancer biology (Fig. **1**).

The cyclic guanosine monophosphate-adenosine monophosphate synthase (cGAS) recognizes free cytoplasmic DNA derived from both self and foreign sources, such as extracellular vesicle DNA. Purple balls represent endocytosis of extracellular vesicle DNA and blue balls represent exocytosis of extracellular vesicle DNA. Activated cGAS then induces the synthesis of cyclic guanosine monophosphate adenosine monophosphate (cGAMP). As the secondary messenger, cGAMP or cyclic dinucleotides (CDNs) bound to the endoplasmic reticulum (ER) adapter protein STimulating INterferon Gene (STING), causing a conformational change, and inducing STING activation. Activated STING then migrates from the ER to the Golgi apparatus. During this process, STING could recruit and activate Tank-binding kinase (TBK1) and inhibitor of nuclear factor-κB (IκBα) kinase (IKK), thereby activating the downstream IRF3 and nuclear factor-κB (NF-kB) signal cascades and inducing the expression of type I interferon (IFN) and inflammatory factors to enhance immune responses.

### 3.1 Tumor Intrinsic *vs.* Immune Cells (APC) Mediated STING Activation Pathway

Immune exclusion in some types of cancer could be explained by understanding the interaction between tumor signaling pathways and immune cells [55, 56]. The differential activation of specific tumor-intrinsic signaling pathways may be responsible for immune exclusion in certain cancers [57]. Cluster of differentiation (CD)155 is a potent immune ligand that regulates the immune response against tumor progression. It interacts with co-stimulatory immunological receptors, which makes it a good candidate to be used as an immunotherapy agent (O’Donnell *et al.*, 2020). Cytotoxic T-lymphocyte-associated (CTLA) 4 and pharmacodynamic (PD) 1 are T cell immune checkpoint molecules that regulate immune responses [58-60]. They are critical in mediating the balance between immune activation and tolerance [61]. Inhibiting these molecules has shown to work as an anti-cancer therapy in various cancer types, underscoring the importance of studying the complex interactions between intrinsic tumors and immune cell pathways. It has been recorded that in PCa, the CTLA response is weaker [62]. This lessened response was characterized by a reduction of CTLA frequency and avidity.

The activated STING pathway produces cytokines and chemokines, which recruit and activate immune cells such as APCs [63, 64]. IFN and other cytokines are produced by the STING pathway, which activates immune cells such as T cells, B cells, and natural killer cells [57, 61]. Enhancing APCs antigen-presenting capabilities leads to a more effective anti-tumor immune response [65, 66].

The activation of the STING pathway can occur through both tumor-intrinsic signals and antigen presentation by antigen-presenting cells (APCs) [67]. Tumor-Intrinsic STING pathway can be activated by cytosolic DNA Damage sensing. Tumor cells often undergo genetic mutations and DNA damage, which can lead to the accumulation of cytosolic DNA fragments. This can happen due to genomic instability, oncogene activation, or other factors [11]. The presence of cytosolic DNA is a danger signal for the immune system. The cGAS (cyclic GMP-AMP synthase) protein is a cytosolic DNA sensor. When it detects aberrant DNA in the cytoplasm, it catalyzes the synthesis of a molecule called cGAMP (cyclic GMP-AMP). cGAMP binds to the STING protein, which is located on the endoplasmic reticulum (ER) membrane. This binding induces a conformational change in STING, leading to its activation. Activated STING then recruits the TBK1 (TANK-binding kinase 1) protein and phosphorylates it. TBK1, in turn, phosphorylates the transcription factor IRF3 (Interferon Regulatory Factor 3). Phosphorylated IRF3 translocates to the nucleus and initiates the transcription of type I interferons (IFN-alpha and IFN-beta) and other pro-inflammatory cytokines [68]. These cytokines are secreted by the tumor cells and act as signaling molecules to alert the immune system about the presence of the tumor [69].

The antigen-presenting cells (APCs), such as dendritic cells, macrophages, and B cells, play a critical role in initiating and modulating immune responses against tumors [70]. When APCs encounter tumor cells, they can phagocytose (engulf) tumor cell fragments or take up antigens released by dying tumor cells [71]. These antigens can include not only proteins but also DNA fragments from the tumor cells. Within the APCs, these tumor-derived DNA fragments can be sensed by cGAS, which then activates the STING pathway as described above. Activation of the STING pathway in APCs leads to the production of type I interferons and other immune signals. These signals help APCs in their function as antigen-presenting cells by promoting the maturation and activation of dendritic cells and enhancing the presentation of tumor antigens to T cells [72].

In summary, the STING pathway can be activated by both tumor-intrinsic signals, such as cytosolic DNA accumulation in tumor cells, and by antigen presentation cells sensing tumor-derived DNA fragments. This activation results in the production of type I interferons and other cytokines, ultimately promoting an immune response against the tumor.

### 3.2 The Mechanism Leading to the Presence of Cytoplasmic Self-DNA in Prostate Cancer

The DNA is exclusively stored in the nucleus and typically should not be in the cytoplasm. Thus, DNA's presence in the cytoplasm signifies cell damage or malfunction [73, 74]. The exact mechanisms leading to cytosolic self-DNA in cancer cells are still under investigation. Gasser *et al.* reported that the cleavage of genomic DNA by DNA structure-specific endonuclease MUS81 leads to the accumulation of cytosolic DNA in PCa cells. This accumulation of PCa DNA is beneficial in PCa autoimmune therapy because it can be used to activate the STING-dependent DNA sensor pathways [75].

The DNA that accumulates in the cytoplasm comes from different sources. One source is the mitochondrial permeability transition pore (mPTP) an opening in the mitochondrial that allows the mitochondrial DNA (mtDNA) to escape from [76]. MtDNA escapes due to the oxidative stress which induces the activation of the cGAS-STING axis. Furthermore, the mitochondria have been shown to be a significant key player in regulating innate immunity thus the STING pathway [77]. Another source is extracellular DNA which accumulates by neutrophil extracellular traps (NETs) [78]. When the nuclear envelope ruptures, the chromatin gets released, and NETs form which have a major impact on cellular pro-inflammatory activity.

## 4 ACTIVATION STRATEGIES OF THE STING PATHWAY

Pre-clinical trials have been initiated to find the proper STING pathway agonist to counteract the tumor cells' inhibitory effect [79]. Different natural and synthetic sources are tested to find the agonist that would yield the highest inflammatory response. The objective is to deliver potent and selective agonists to create an adequate inflammatory response in the TME, allowing for a more significant response to immunotherapy and overcoming acquired resistance.

### 4.1 Sting Agonists

#### 4.1.1 Cyclic Dinucleotides (CDNs)

Cyclic dinucleotides such as 2'3'-cGAMP, 3'3'-cGAMP, cyclic dipolyguanosine phosphate (cdi-GMP), and cyclic dipolyadenosine phosphate (c-di-AMP) are known to target STING proteins and enhance antigen presentation and immune response strength. 2'3'-cGAMP, a second messenger in mammals, activates the STING pathway and is transformed by calcitonin-related peptides [80]. Several analogs to 2'3'-cGAMP, such as 3'3'-cGAMP, C-di-GMP, and C-di-AMP, have been found in bacteria and archaea, which bind to STING proteins [81]. As STING is mainly found in the endoplasmic reticulum, CDNs need to penetrate cell membranes for their binding and subsequent function [82]. Natural CDNs, however, are negatively charged (containing two phosphate groups) and highly hydrophilic; therefore, they cannot easily cross cell membranes. In addition, natural CDNs are susceptible to phosphodiesterase hydrolysis leading to their inactivation [83]. Therefore, systemic administration of CDNs may lead to rapid degradation and loss of anti-tumor activity. Initially, CDNS was demonstrated to have anti-tumor properties by injecting C-di-GMP into cancer models to stimulate the immune system [84].

Overall, the *in vivo* application of CDNs requires drug delivery systems to prevent hydrolysis and improve targeting accumulation in tumors and cellular uptake for STING activation. ADU-S100 (MIW815) was the first CDN agent to be used in cancer immunotherapy clinical trials, with enhanced stability and lipophilicity [85]. It was shown that treatment with ADU-S100 significantly reduced tumor growth in mice. It also enhanced systemic immune responses to combat far- away metastasis and prolong the immunologic memory in B16 melanoma, CT26 colon cancer, and 4T1 breast cancer models [86]. MK-1454 is an advanced-performance synthetic CDN derivative. Patients with advanced solid tumors or lymphomas injected with MK-1454 alone or in combination with ICI pembrolizumab showed good activity and safety in a phase I study (NCT03010176) [87]. In addition, intra-tumoral injection of MK-1454 alone or in combination with pembrolizumab was evaluated in patients with metastatic or incurable recurrent HNSCC (NCT04220866). A recent study has shown that injecting STING agonist (ADU-S100) into mice with bilateral PCa tumors had significant tumor regression [88].

#### 4.1.2 Non-nucleotidyl Small Molecule STING Agonists

A STING agonist, DMXAA, is derived from flavone 8-acetic acid, which was initially used in preclinical trials as a vascular disrupter [89]. DMXAA interacts directly with STING and has been shown to potentiate anti-tumor effects in mice models [90]. DMXAA (ASA404) was studied in combination with carboplatin and paclitaxel in a single-arm phase II study in patients with advanced NSCLC. The combination was well-tolerated and resulted in no cardiac adverse events or other side effects, demonstrating improvements in efficacy variables and survival of advanced NSCLC [91]. In the following phase III clinical trial, no difference was found between the DMXAA treatment and placebo groups regarding survival and progression-free survival for patients with advanced NSCLC [92]. Unfortunately, human STING cannot be activated by DMXAA. In contrast, preclinical studies in mice models demonstrated that DMXAA induces significant innate immune responses and potent anti-tumor effects in mice [86]. DMXAA can induce the production of NF-κB in tumor cells [93] and endothelial cells [94]. The possibility is that DMXAA could stimulate phosphorylated IRF dimer translocation to the nucleus while also activating NF-κB targets [95].

Analogs based on amido benzimidazole (ABZI) were synthesized to increase systemic delivery as they can connect to the C-terminal domain of STING and increase the binding affinity [96]. From linked ABZIs, the representative one is diABZI, which strongly improved STING affinity and stimulated IFN production in human PBMCs. The administration of diABZI to mice with CT26 colorectal cancers significantly reduced tumor growth and improved survival, with 80% of the mice being tumor-free [97]. Compound 3 is an ABZI-based compound that can activate STING, produce IFN-β, inhibit CT26 colorectal tumor growth in mice, and improve survival by increasing T cell immune responses [97]. Moreover, MSA-2, an orally available small-molecule STING agonist, has been shown to promote tumor regression and stimulate IFN- β production in multiple syngeneic mouse tumor models (Table **2**) [98]. 

#### 4.1.3 Different Effects of STING Agonists

Different STING agonists have gained significant attention for their potential use in cancer immunotherapy and as adjuvants in vaccine development. Here are some of the different effects of STING agonists. In summary, different STING agonists have diverse effects, including the induction of antiviral immunity, enhancement of anti-tumor immunity, adjuvant effects in vaccines, and the potential for inflammatory responses.

STING agonists can induce antiviral immunity such as hepatitis B virus [101]. One of the primary roles of the STING pathway is to detect viral infections. When STING is activated by viral DNA or other viral components, it triggers the production of type I interferons and pro-inflammatory cytokines. These cytokines help to combat viral infections by inhibiting viral replication, activating immune cells, and enhancing the immune response against the virus. Secondly, STING agonists can also enhance anti-tumor immunity. STING agonists have shown promise in cancer immunotherapy. When tumors release DNA or other molecules that activate the STING pathway, it can lead to an anti-tumor immune response for cancer immunotherapy [102]. Activation of STING in tumor cells or antigen-presenting cells (APCs) results in the production of type I interferons and cytokines. Type I interferons help recruit and activate immune cells, such as cytotoxic T cells and natural killer (NK) cells, to target and destroy tumor cells. STING agonists can also promote the maturation and activation of dendritic cells, which are essential for presenting tumor antigens to T cells and initiating an adaptive immune response against the tumor. Thirdly, STING agonists can be used as adjuvants for vaccines: STING agonists can be used as adjuvants in vaccine development. When included in a vaccine formulation, they can enhance the immune response to the vaccine antigen. STING agonists stimulate the production of type I interferons and cytokines, which activate immune cells and improve the antigen-presenting capacity of APCs. This results in a more robust and durable immune response to the vaccine, making it more effective in protecting against infections. Last but not least, STING agonists can induce inflammatory Responses. Excessive or prolonged activation of the STING pathway can lead to harmful inflammatory responses. In autoimmune diseases such as monogenic autoinflammation and lupus, the STING pathway can be dysregulated, leading to chronic inflammation. The inappropriate activation of the STING pathway can contribute to inflammatory disorders and tissue damage [101, 103].

### 4.2 Bacterial Vectors

Aside from the aforementioned STING agonists, bacterial vectors are being studied as novel delivery techniques for STING agonists to transform into target cells. For example, SYNB1891 is a vector localized to the tumor microenvironment and expresses the enzymes that produce c-di-AMP. Intratumoral injection of SYNB1891 into mice with B16.F10 melanoma tumors resulted in the production of IFNs and tumor regression [104]. Furthermore, the anti-tumor efficacy of intratumoral SYNB1891 is being evaluated in an ongoing phase I clinical trial of patients with advanced/metastatic solid tumors and lymphoma (NCT04167137).

### 4.3 Radiotherapy

Typically, DNA double-strand breaks (DSBs) are viewed as the deadliest type of DNA injury and a leading factor in cell death, resulting from ionizing radiation (IR) exposure during radiotherapy. Signaling cascades originating in the cytoplasm as a result of oncoproteins like RTKs and RAS activate the conventional pathways for repairing double-strand DNA breaks, specifically non-homologous end joining (NHEJ) and homologous recombination (HR). Directly inhibiting these established pathways, such as through the use of DNA-PKcs or ATM inhibitors, is highly likely to impede double-strand DNA repair in both normal and tumor cells [105]. The accumulating tumor pathological DNA in the cytoplasm can activate the cGAS-STING signaling pathway, inducing innate immunity and promoting adaptive immunity [106, 107]. Recent research has demonstrated that the radiation treatment and the consequent anti-tumor efficacy are closely connected to the activation of the cGAS-STING pathway and the production of IFN [108, 109]. Deng *et al.* demonstrated that mice with a STING or cGAS deficit were not activated by a considerable dose of radiation, and IFN could not induce DCs even after radiotherapy. In addition, they demonstrated that the cGAS-STING-dependent cytoplasmic DNA sensing pathway is required for a radiation-activated adaptive anti-tumor immune response. Nonetheless, the conducted radiation dose determines whether radiotherapy causes cGAS-STING- mediated anti-tumor effects or not. Radiation dose is crucial for STING pathway activation. It has been proven that low doses of radiation may elicit insufficient biological responses, whereas high doses of radiation may result in harmful side effects [110].

## 5 COMBINATION OF RADIOTHERAPY AND STING AGONISTS

Radiation-induced DNA damage can activate the cGAS-STING pathway and induce T-cell responses [100], and the combination of radiotherapy with STING agonists causes a more potent anti-tumor effect. Xue *et al.* reported that the STING agonist diABZI increased the radiosensitivity of NSCLC cells to irradiation by activating the cGAS-STING pathway and promoting apoptosis [111]. Moreover, a type of liposome nanoparticle loaded with cGAMP in combination with radiotherapy could suppress lung metastasis of 4T1 breast cancer [112]. Moreover, Luo *et al.* demonstrated that the augmentation of T cell response by radiotherapy and nano vaccine (mixture of an antigen and polymer nanoparticle) depends on the STING pathway. In mice with a STING mutation or deficiency, the anti-tumor efficacy of this method is significantly decreased [112]. In a mouse model of homologous melanoma or neuroblastoma, the nano-vaccine, in conjunction with radiation-stimulated DCs and effector T cells, resulted in evident tumor reduction and specific anti-tumor immune memory [113, 114]. In a mouse model of lung metastasis, a form of liposome nanoparticle loaded with cGAMP in combination with radiation-induced the production of IFN in APC, resulting in strong anti-tumor feedback.

### 5.1 Radiotherapy (RT): Dosage and Fractions

The cGAS-STING pathway increases the immune system's anti-tumor response to RT [115]. When the cGAS-STING pathway is activated, it detects damaged DNA, triggering the induction of IFN response. One effective method to activate the STING pathway is to use hypofractionation [116]. It activates STING by administering larger doses of RT in fewer fractions.

However, the optimal RT dose that activates IFN is moderately high, between 8-12 Gy [117]. Extremely high doses of 20-30 Gy in 1 fraction potentially could lead to the inhibition of negative feedback by Trex 1 exonuclease [118]. Thus, reducing the accumulation of cytoplasmic DNA and preventing the activation of the cGAS signaling pathway. The effectiveness of the radiation-induced anti-tumor immune response is terminated when cGAS activation signaling is halted.

The RT dose and fractionation schedule is critical in optimizing the immune system's anti-tumor response [119, 120]. Combining RT regimens with immunotherapy, effectively induces clinically relevant anti-tumor immunity. Different strategies are being investigated to determine the most effective consideration of particle type, dose, and fractionation. For example, in a phase II study, researchers studied the optimal RT dose and fractionation when combining ipilimumab immunotherapy in non-small cell lung cancer [111, 121].

## 6 ACTIVATION OF STING PATHWAY IN PROSTATE CANCER

The inhibition of the STING pathway in PCa plays a significant factor in limiting the current conventional therapy [122]. On the other hand, STING has been shown to play a role in PCa tumor rejection [15]. Thus, finding ways to activate STING in PCa target therapy is essential. Studies have documented strong evidence of the killing of PCa cells by STING pathway activation [122]. In an *in-vitro* PCa-lymphocyte co-culture model, the study investigated whether the combination of STING agonist (ADU-S100) and Interleukin-15 (IL-15) increases the therapeutic potential in targeting PCa cells. They demonstrated that this combination induces NK cells, and results in a considerably higher PCa cell death than either drug alone [123]. Another study used transgenic adenocarcinoma of the mouse prostate model (TRAMP-C2) and evaluated STING agonist cyclic di-GMP with immune checkpoint blockers (CTLA-4, PD-1, and 4-1BB). They showed that STING/checkpoint therapy increased T cell infiltration and promoted the cure of 75% of mice with bilateral TRAMP-C2 [124]. cGAS/STING have also been recoded to remodel the TME to increase T cells in PCa [125-128]. The knocking out of STING in the murine model, limited the effectiveness of anti-PD1 and chemo-hormonal therapy. Furthermore, the loss of STING reduced T cell penetration of TME.

Using the STING pathway has been shown to clinically improve the benefits of immunotherapy. In a cohort study, scientists combined the STING pathway and anti-PD1 blockade to treat resistant prostate cancer patients. It resulted in an improved prostate-specific antigen progression-free survival [125].

Recent studies have described TMEM173, a novel STING protein, as a potential biomarker that correlates with the growth of PCa and progression [126]. PCa tissue has been shown to have a significant elevation of TMEM173 production. Also, a new method of STING activation in which the magnetic PEGylated manganese-zinc ferrite nanocrystals (PMZFNs) have been shown to increase the effectiveness of ICD against PCa [121]. PCa-associated Speckle-type POZ protein (SPOP) mutation, a DNA stabilizing protein, has been shown to stabilize STING which reprograms the TME, and strengthens the immune response in inhibiting PCa growth [127]. Bromodomain and extra-terminal (BET) domain inhibition (BETi) have been shown to remodel PCa TME and increase immune infiltration through activating the STING pathway [128].

## CONCLUSION

Managing PCa has been challenging, but recent advancements in cancer research have provided a glimmer of hope for patients in the fight against it. The Stimulator of Interferon Genes (STING) pathway has been shown to boost the immune system's ability to recognize and eliminate malignant cells. One mechanism for STING is the production of IFN, which works to enhance immunotherapy effectiveness against cancer.

Scientists have classified tumors based on their capacity to trigger immune responses. PCa falls into the “cold” tumor category, making it difficult to treat with immunotherapy. However, by activating the STING pathway, PCa could turn into a “hot” tumor, making it a better target for immunotherapy.

The combination of RT and STING has been demonstrated to be effective against cancer. Further research is necessary to determine the efficacy of STING's activation in cancer treatment and the side effects of such a therapy. Nonetheless, this breakthrough in the treatment of cancer offers hope for patients.

## Figures and Tables

**Table 1:** Mechanisms that contribute to the immune response in PCa.

**Factor**	**Mechanism of Immune Resistance in PCa**	**References**
Tumor Mutational Burden (TMB)	Decreased somatic tumor mutational burden (TMB) leads to reduced neoantigen expression and anti-tumor response.	[32]
Interferon Pathway	The loss of IFN seen in PCa causes a suppression of anti-cancer response and promotes bone cell activation.	[26]
PTEN	Prostate tumors that are deficient in PTEN exhibit higher densities of regulatory T cells, suggesting an amplification of immunosuppressive factors within the TME.	[33]
Major Histocompatibility Complex (MHC) Class I Expression	Androgen deprivation therapy (ADT) increases castration resistance, resulting in immunological tolerance to PCa antigens.	[34, 35]
Myeloid-derived suppressor cells (MDSC)	Accumulation of myeloid-derived suppressor cells (MDSCs) immunosuppressive population within the TME.	[27]
Androgen Receptor Signaling	MHC-I activates T cells and promotes the destruction of target cells. Both metastatic PCa cell lines and clinical specimens showed loss of MHC-I expression.	[36]

**Table 2:** Summary of CDN and non-CDN STING agonists.

**Agonist**	**Structure**	**Preclinical Characterization**	**Clinical Trial**	**References**
2’3’-cGAMP	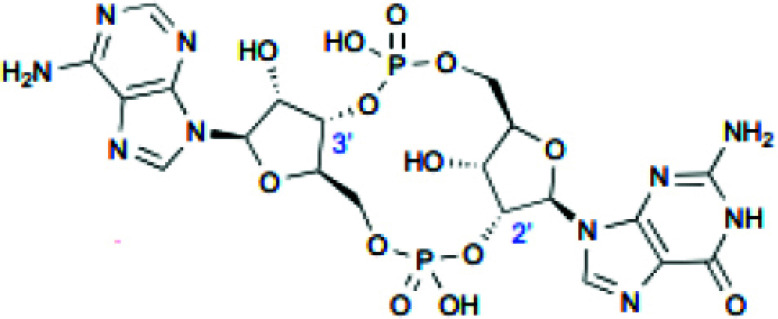	In the MC38 syngeneic mouse tumor model, radiation (10 μg, IT) enhanced antitumor activity.	None	[99]
ADU-S100	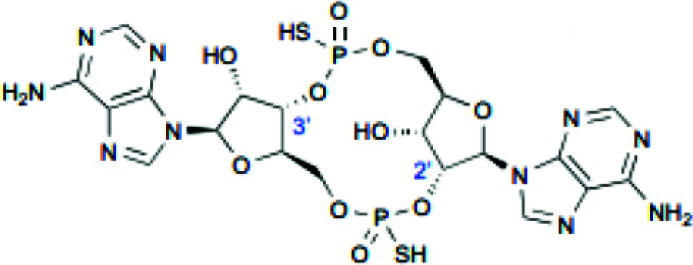	Tumor growth was reduced in mice bearing B16 and F10 tumors (25 μg, IT) and 4T1 and CT26 mammary carcinomas (50 μg, IT).	Phase 1: NCT02675439 Phase 1b: NCT03172936 Phase 2: NCT03937141	[86]
MK-1454	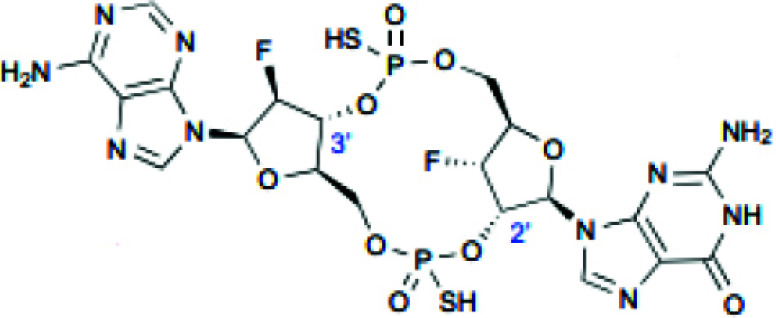	MC38 syngeneic mouse tumor model (20 μg, IT) showed inhibited tumor growth and elevated IFN-β, IL-6, and TNF-α levels.	Phase 1: NCT03010176 Phase 2: NCT04220866	[87]
DMXAA	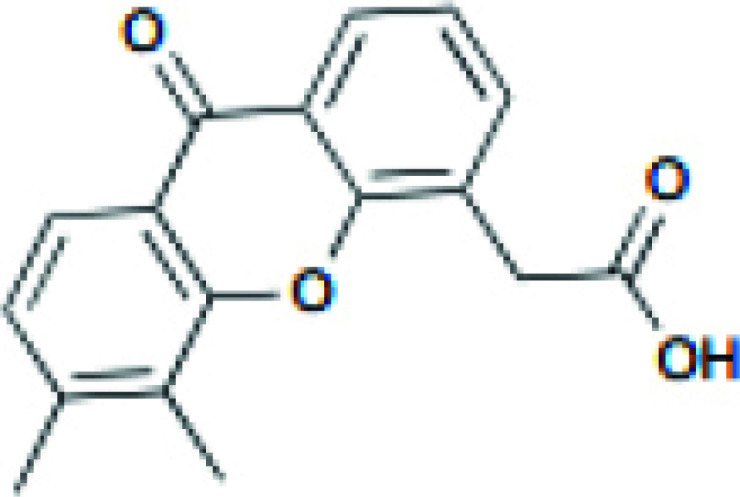	Tumor size reduction in B16.SIY syngeneic mouse model (500 μg, IT)	18 clinical trials, reviewed in (102)	[100]
di-ABZI-3	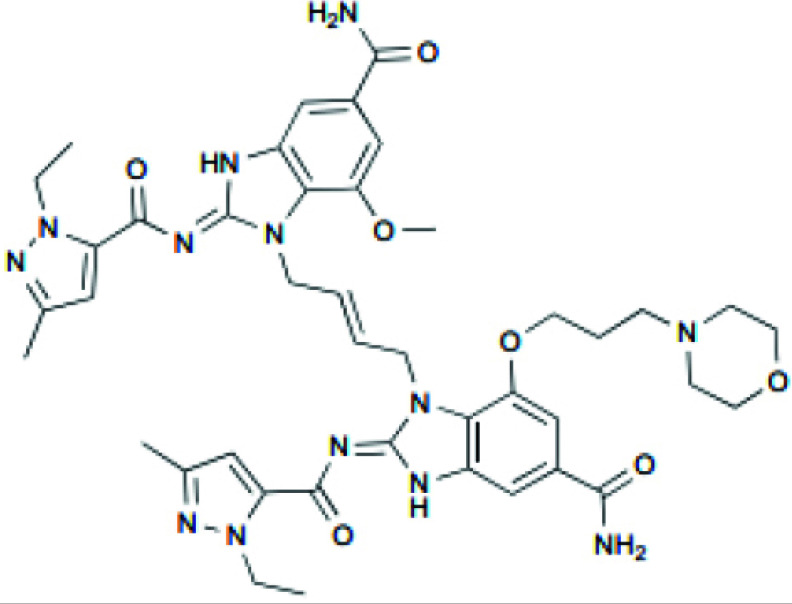	Induced IFN-β in human PBMCs; tumor growth inhibition in CT-26 syngeneic mouse model in BALB/c mice (1.5 mg/kg, IV)	None	[97]
MSA-2 O	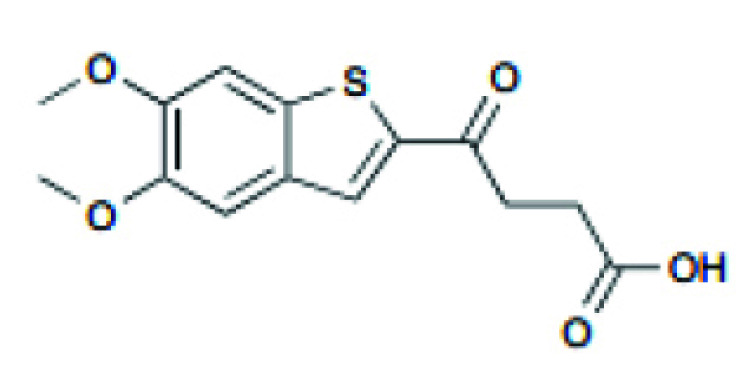	Tumor growth inhibition in MC38 mouse model (450 μg, IT; 50 mg/kg, SC; 60 mg/kg, PO)	None	[98]

## LIST OF ABBREVIATIONS

PCa: Prostate Cancer
STING: Stimulator of Interferon Genes
TME: Tumor Microenvironment
ICIs: Immune Checkpoint Inhibitors
